# A large-scale polygenic risk score analysis identified candidate proteins associated with anxiety, depression and neuroticism

**DOI:** 10.1186/s13041-022-00954-3

**Published:** 2022-07-23

**Authors:** Bolun Cheng, Xuena Yang, Shiqiang Cheng, Chun’e Li, Huijie Zhang, Li Liu, Peilin Meng, Yumeng Jia, Yan Wen, Feng Zhang

**Affiliations:** 1 Key Laboratory of Trace Elements and Endemic Diseases, Collaborative Innovation Center of Endemic Disease and Health Promotion for Silk Road Region, School of Public Health, Health Science Center, Xi’an Jiaotong University, 76 Yan Ta West Road, 710061 Xi’an, People’s Republic of China; 2 Key Laboratory for Disease Prevention and Control and Health Promotion of Shaanxi Province, Xi’an, People’s Republic of China

**Keywords:** Polygenic risk score, Protein quantitative trait loci, Neuroticism, Depression, Anxiety

## Abstract

Psychiatric disorders and neuroticism are closely associated with central nervous system, whose proper functioning depends on efficient protein renewal. This study aims to systematically analyze the association between anxiety / depression / neuroticism and each of the 439 proteins. 47,536 pQTLs of 439 proteins in brain, plasma and cerebrospinal fluid (CSF) were collected from recent genome-wide association study. Polygenic risk scores (PRS) of the 439 proteins were then calculated using the UK Biobank cohort, including 120,729 subjects of neuroticism, 255,354 subjects of anxiety and 316,513 subjects of depression. Pearson correlation analyses were performed to evaluate the correlation between each protein and each of the mental traits by using calculated PRSs as the instrumental variables of protein. In general population, six correlations were identified in plasma and CSF such as plasma protease C1 inhibitor (C1-INH) with neuroticism score (r = − 0.011, *P* = 2.56 × 10^− 9^) in plasma, C1-INH with neuroticism score (r = -0.010, *P* = 3.09 × 10^− 8^) in CSF, and ERBB1 with self-reported depression (r = − 0.012, *P* = 4.65 × 10^− 5^) in CSF. C1-INH and ERBB1 may induce neuroticism and depression by affecting brain function and synaptic development. Gender subgroup analyses found that BST1 was correlated with neuroticism score in male CSF (r = − 0.011, *P* = 1.80 × 10^− 5^), while CNTN2 was correlated with depression score in female brain (r = − 0.013, *P* = 6.43 × 10^− 4^). BST1 and CNTN2 may be involved in nervous system metabolism and brain health. Six common candidate proteins were associated with all three traits (*P* < 0.05) and were confirmed in relevant proteomic studies, such as C1-INH in plasma, CNTN2 and MSP in the brain. Our results provide novel clues for revealing the roles of proteins in the development of anxiety, depression and neuroticism.

**Supplementary Information:**

The online version contains supplementary material available at 10.1186/s13041-022-00954-3.

## Introduction

Neuroticism is a complex health-related personality factor that includes anxiety, moodiness, worrying, and negative emotions, and people affected by neuroticism feel, notice, and report more distress, symptoms and pain [1]. Generalized anxiety disorder is characterized by chronic, pervasive anxiety and worry accompanied by nonspecific physical and psychological symptoms, including restlessness, fatigue, difficulty concentrating, irritability, muscle tension, or difficulty sleeping [2]. Depression often presents with low self‑esteem, low mood, anhedonia, feeling of worthlessness, fatigue, sense of rejection and guilt, suicidal thoughts, among others [3]. Anxiety and depression are common psychiatric disorders with lifetime prevalence of 12.9% (reported in 2014) [4] and 16.2% (reported in 2003) [5] respectively. Neuroticism is an important contributing factor for both anxiety and depression [6]. Recent regression analyses concluded that neuroticism significantly predicted depression and anxiety [7]. Nagel et al. performed Mendelian randomization analysis and observed bidirectional associations between neuroticism and depression [8]. Although many genetic variants associated with neuroticism and anxiety/depression were identified, the relationships between these traits at the protein level remains elusive [8].

Changes of protein abundance in human brain were associated with psychiatric disorders and neurodegenerative diseases [9, 10], involving multiple regulatory mechanisms in transcription and translation, such as miRNA control and ubiquitin proteasome dependent degradation [11, 12]. Felger et al. identified the clusters of cerebrospinal fluid (CSF) inflammatory markers that were correlated with depressive symptom severity [13]. Wang et al. integrated multiple proteomes including cortex, CSF and serum in Alzheimer’s disease (AD), and identified 37 proteins emerged as potential AD biomarker across these three tissues [14]. Studies have shown that the proteins involved in brain, CSF and plasma were significantly different in people with mental disorders than in the general population [15, 16]. Therefore, a systematic study is needed to explore the relationships between anxiety, depression, and neuroticism with proteins in brain, CSF and plasma from a genetic perspective.

Although historically research has focused on transcription as the central governor of protein expression, protein translation is now increasingly being recognized as a major factor for determining protein levels within cells [17]. SNPs in coding region or non-coding region may be associated with expression quantitative trait locus (eQTL) or altered protein quantitative trait loci (pQTL) [18]. Many eQTLs have been identified to be associated with the mRNA expression of psychiatric disorders [19, 20]. However, the mRNA expression of many genes is poorly correlated with protein levels, in part due to the influence of many post-transcriptional factors such as protein translation and degradation [21]. Compared with eQTL, pQTL mapping analysis showed that pQTL could provide more effective insights into the effects of genetic variation [22]. Increasing evidence also suggested that impaired mRNA translation is a common feature found in numerous complex diseases [23, 24]. Thus, pQTL may play a key role in the post-transcriptional regulation mechanism of complex disease-related proteins.

Genome-wide association studies (GWASs) have identified multiple risk variants for complex diseases [8, 25]. Nevertheless, to what extent the risk variants of complex diseases can lead to cumulative risk of individual remains largely unknown. Polygenic risk score (PRS) was proposed to solve this dilemma, which reflects the sum of all known risk loci [26]. PRS is an individual-level score calculated based on the number of risk variants, and weighted by SNP effect sizes derived from an independent large-scaled discovery GWAS [26]. The effect sizes of multiple SNPs are combined into a single aggregate score that can be used to predict the risks of human diseases [27]. Recently, PRS has shown promise in investigating the association between different psychiatric disease [28]. Lin et al. tested the ability to predict brain disorders in postmortem expression datasets and clinical cohorts, and found that PRS^cis−eQTL^ scores were associated with late-life depression [29].

The present study systematically analyzed the association between protein in brain, CSF and plasma with neuroticism, anxiety and depression. The PRS scores of proteins in different tissues were calculated using the genotype data from the UK Biobank cohort, respectively. Pearson correlation analyses were then performed to investigate whether each protein was correlated with neuroticism, anxiety and depression by using calculated PRSs as the instrumental variables of protein. Our study may provide new insights into the application of pQTL data, and highlight the significant impact of proteins on the risks of neuroticism, anxiety and depression.

## Methods

### Neuroticism, anxiety and depression phenotypes in the UK Biobank cohort

The phenotypic and genotypic data used here were derived from the UK Biobank, which has recruited 502,656 participants aged between 40 and 69 years, and conducted a large prospective cohort study from 2006 to 2010 [30]. The UK Biobank has collected a large collection of phenotypic, health-related information for each participant, including biometric and physical measurements, lifestyle indicators and genome-wide genotyping data. The present study accessed health-related records of each participant, including age, sex, smoking and alcohol use, Townsend deprivation index (TDI), body mass index (BMI), and education scores from screenshot question or verbal interview within assessment center. Neuroticism (data fields: 20,217) was defined based on Eysenck personality questionnaire (EPQ) and revised short form (FPQ-R-S) [31]. Anxiety (data fields: 20,421 and 20,420) was defined based on general anxiety disorder (GAD-7) and composite international diagnostic interview short-form (CIDI-SF), while depression (data fields: 20,002, 20,126 and 20,544) was defined based on patient health questionnaire (PHQ-9) and CIDI-SF [32, 33]. In this study, neuroticism used symptom scores, while anxiety and depression used both case-control status and symptom scores. For the case-control phenotype, PHQ score ≤ 5 and GAD score < 5 were defined as the control cut-off for depression and anxiety, respectively. Ethical approval of UK Biobank was granted by the National Health Service National Research Ethics Service (reference 11/NW/0382). Neuroticism, anxiety and depression score were mean-centered and normalized to one standard deviation (SD) before further analysis. The detailed phenotype definitions of neuroticism, anxiety and depression in this study are shown in Additional file 1.

### UK Biobank genotyping, imputation and quality control

Genome-wide genotyping was conducted in 489,212 participants with 812,428 SNPs using either the Affymetrix UK BiLEVE Axiom or Affymetrix UK Biobank Axiom array. Imputation was conducted by IMPUTE2 using the reference panel of the Haplotype Reference Consortium, 1000 Genomes and UK10K projects [30]. The SNPs with high linkage disequilibrium (*r*^2^ > 0.5) were removed to select high-quality SNPs. 488,377 participants and 805,426 SNPs were kept after applying quality control measures. The researchers provided a list of 409,728 participants who self-report ethnicity as “British” and who have very similar genetic ancestral backgrounds according to the PCA. This set of individuals was referred as the “white British ancestry subset” (UK Biobank field ID: 21000) [30]. After removing participants who reported inconsistencies between self-reported gender and genetic gender, as well as whom missing covariate information, 376,806 participants were retained for further analysis. Details of the array design, genotyping, and quality control procedures were published elsewhere [30].

### Polygenic risk score datasets of neurological proteins

2678 pQTLs of 70 proteins in brain, 11,605 pQTLs of 152 proteins in plasma and 33,253 pQTLs of 217 proteins in CSF were collected from the proteome atlas of neurological disorders (https://www.ncbi.nlm.nih.gov/pmc/articles/PMC8521603/) [34]. Briefly, protein samples from 1537 participants included three tissue types: CSF (collected from living individuals), plasma (collected from living individuals) and brain (collected from fresh frozen human parietal lobes). The proteomics data were processed using SomaDataIO (v1.8.0) and Biobase (v2.42.0). Proteins were mapped to UniProt identifiers and Entrez Gene symbols. Ensembl gene IDs and genomic position mapping was performed using gencode version 30 [34]. Based on the original research [34], QC on both proteins and samples were described as follows. The protein level QC, starting from 1305 proteins; after step-1, Limit Of Detection VS 2-StDeviation, 807 CSF, 1301 plasma, 1109 brain proteins were kept with a pass-rate ≥85%; after step-2, given Max Difference of Scale Factor < 0.5, 749 CSF, 956 plasma, 1107 brain proteins were kept; after step-3, given Coefficient of Variation (of calibrator) < 0.15 and step-4, given IQR, sum(outliers) < 15%, 746 CSF, 955 plasma, 1106 brain proteins were kept. After step-5, 713 CSF, 931 plasma, 1079 brain proteins that shared by < 30 samples, < 10 samples, and < 21 samples (shared by ~ 80% of the subject outliers) were kept, respectively. The sample level QC, the proteomics from 1300 CSF, 648 plasma and 459 brain samples were profiled within each tissue. 971 CSF, 636 plasma and 458 brain samples were from unique donors in proteomics data. 965 CSF, 633 plasma and 450 brain samples were kept with available genotyping array data. 875 CSF, 561 plasma and 426 brain samples were kept with a European ancestry after adjusting for principal components. Moreover, 853 CSF, 542 plasma and 400 brain samples were kept that were not closely related with one another (PI_HAT < 0.05) after checking identity by descent. Finally, 835 CSF, 529 plasma and 380 brain samples remained by passing both the genotype and protein data QC. After removing low-quality SNPs, genotype imputation was performed using the Impute2 program with haplotypes derived from the 1000 Genomes Project. SNPs with an info-score quality of less than 0.3 reported by Impute2, with a MAF < 0.02 or out of HWE were removed [34]. A total of 14,059,245 imputed and directly genotyped SNPs were used for final analyses. To test the association between genetic variants and protein levels, a linear regression with additive model was performed using age, sex, principal component factors from population stratification and genotype platform as covariates [34]. The detailed information of sample collection, aptamer-based proteomics, proteomic and genomic data QC process, pQTL identification, and annotation of pQTL were described elsewhere [34].

### PRS calculation of pQTL in the UK Biobank cohort

The linkage disequilibrium independent SNPs (*r*^2^ > 0.5) were first pruned for each protein using PLINK 2.0. According to the standard approach, PLINK 2.0 was used to calculate the PRS of each study subject for each protein using linkage disequilibrium independent SNPs and individual genotype data from the UK Biobank (http://www.cog-genomics.org/plink/2.0/) [35]. Briefly, we set *PRS*_*n*_ denotes the PRS value of pQTL for the *n*th subject, defined as:

$${PRS}_{n}={\sum }_{i = 1}^{l}{E}_{i}{D}_{in}$$

where *l* denotes the total number of pQTL associated SNPs; *E*_*i*_ denotes the effect size of significant pQTL associated SNP *i*; *D*_*in*_ denotes the dosage of the risk allele of the *ith* SNP for the *nth* individual (0 is coded for homozygous protective genotype, 1 for heterozygous and 2 for homozygous polymorphic genotypes).

### Covariates in regression models

Alcohol use frequency/week, smoking frequency/day, body mass index (BMI), education score and Townsend deprivation index (TDI) were used as the covariates in regression models to improve the accuracy of our analysis. The association between smoking with depression and anxiety was found to be bidirectional, with occasional smoking initially used to alleviate symptoms, but in fact worsening them over time [36]. A longitudinal follow-up study suggested that alcohol consumption as a risk factor for anxiety and depression [37]. Torgersen et al. found a shared genetic structure between neuroticism and BMI, of which 61 of the shared loci with BMI are novel for neuroticism [38]. Recently, we found the relevance of the TDI to psychiatric disorders such as anxiety and depression, and identified several candidate genes interacting with the TDI [39]. TDI (data field: 189) was calculated immediately prior to participant joining UK Biobank, based on the preceding national census output areas. Each participant is assigned a score corresponding to the output area in which their postcode is located. Education scores (level 1–5 variables, representing the level of education from low to high) were constructed by mapping each major educational qualification in UK Biobank (data field: 6138) to an International Standard Classification of Education (ISCED) category [40].

### Statistical analysis

The neuroticism score, anxiety score and depression score were firstly adjusted for top 3 principle components of population structure (PC1-PC3), sex, age, alcohol use frequency/week, smoking frequency/day, TDI, BMI, and education score as covariates using linear regression models. The self-reported anxiety and self-reported depression were firstly adjusted for the same covariates as above using logistic regression models. The residuals from regression models were then used as the phenotypic values for Pearson correlation analysis. Pearson correlation analysis was then performed to evaluate the correlation between each protein and each of the phenotypes by using calculated PRS as the instrumental variables of protein. The R software (version R 3.5.3) was used to conduct Pearson correlation analysis. The significant association thresholds should be *P* < 0.05/(number of independent protein) after strict Bonferroni correction. There were 70, 152, and 217 independent proteins in brain, plasma, and CSF, respectively. Therefore, *P* value thresholds were set at 7.14 × 10^− 4^ for brain, 3.29 × 10^− 4^ for plasma, and 2.30 × 10^− 4^ for CSF.

### Validation of candidate proteins in proteomic studies

The association signals of proteins and pQTLs from previous proteomic studies were used to validate our results. Firstly, relevant proteomic studies were searched to verify the common proteins associated with anxiety, depression, and neuroticism in our study. In detail, a comprehensive literature search was conducted in PubMed up until December 1, 2021. The keywords in the search strategy included (proteomic[Title]) AND (anxiety[Title/Abstract]), (proteomic[Title]) AND (depression[Title/Abstract]), and (proteomic[Title]) AND (neuroticism[Title/Abstract]). The pQTL data in QTLbase were subsequently used to validate the significant relevant pQTLs in our study. QTLbase organizes and compiles genome-wide QTL summary statistics for many human molecular traits across over 70 tissue or cell types [18]. The database comprises tens of millions significant genotype-molecular trait associations under different conditions. Search by trait in QUERY option was used to verify pQTLs in brain tissues according to protein name and the corresponding EntrezGeneSymbol.

## Results

### Descriptive characteristics of study samples

The descriptive characteristics of participants in this study are presented in Table 1. There were 120,729, 316,513 and 255,354 study subjects for neuroticism score, depression score and anxiety score, respectively. Correlation matrix among covariates and neuroticism, anxiety, and depression score are presented in Additional file 2.

**Table 1:** Descriptive characteristics of participants in this study

	No.	Mean ± SD	Range
Age, years	376,806	56.99 ± 7.93	39, 73
Sex			
Male	202,434		
Female	174,372		
Neuroticism score	120,729	4.12 ± 3.30	0, 12
Depression score	316,513	3.92 ± 3.37	0, 27
Anxiety score	255,354	2.40 ± 3.53	0, 27
TDI	376,352	− 1.56 ± 2.93	− 6.26, 10.88
BMI	370,229	27.42 ± 4.74	12.80, 68.40
Alcohol use frequency/week	302,658	10.53 ± 10.20	0.00, 483.00
Smoking frequency/day	320,160	6.53 ± 10.67	0.00, 140.00
Education score	376,801	3.38 ± 1.51	1.00, 5.00

Age was described as mean standard deviation (SD); *TDI* Townsend deprivation index, *BMI* body mass index, *No.* number of samples

### Disease/trait-associated proteins in general population

**Fig. 1:**
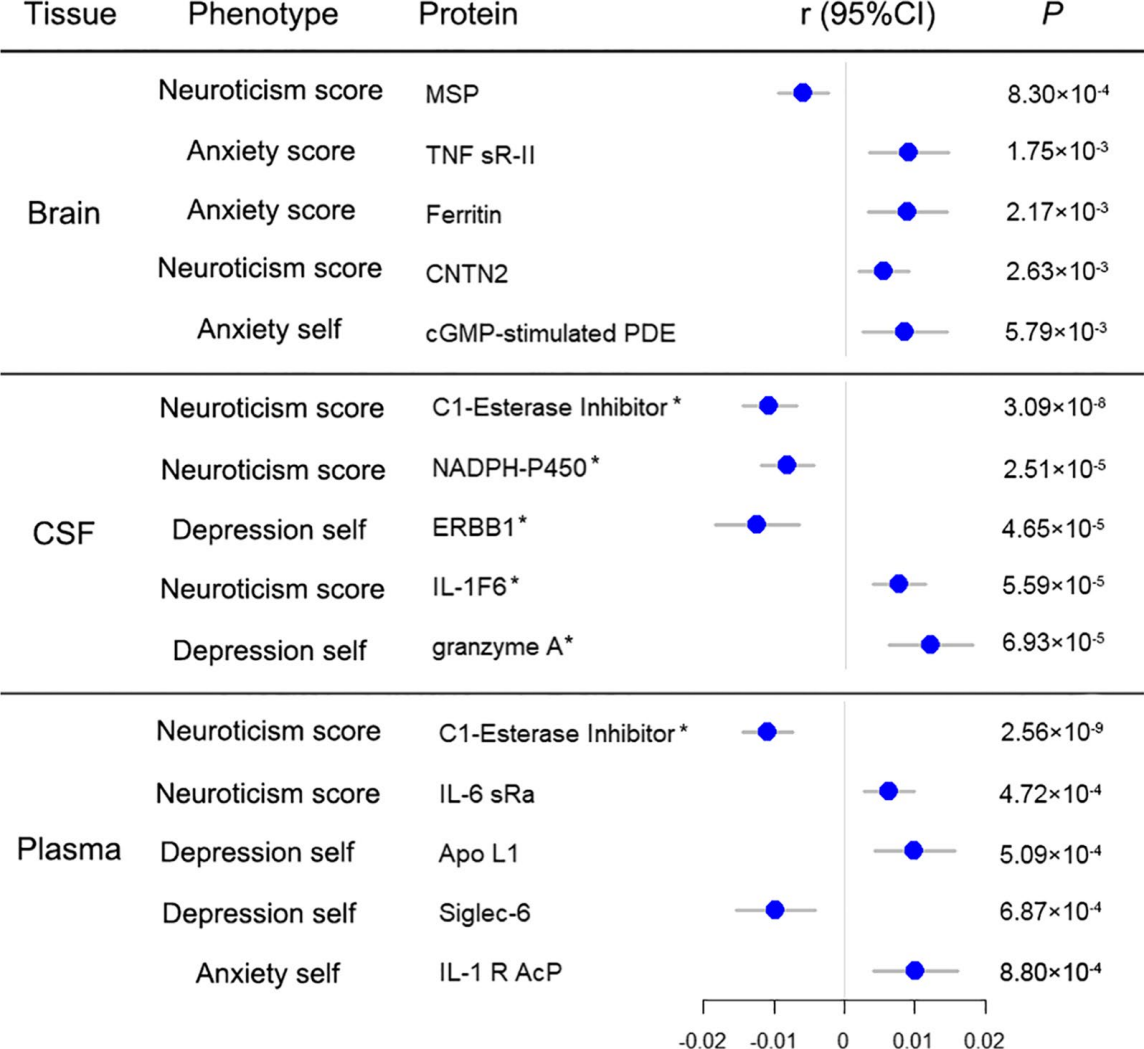
The top 5 correlations between protein and anxiety, depression and neuroticism in general population. Dots indicate protein specific betas; Horizontal lines represent 95% CI. * indicates the protein that has reached the significant threshold of Bonferroni correction (7.14 × 10^− 4^ for brain, 3.29 × 10^− 4^ for plasma, and 2.30 × 10^− 4^ for CSF). self, self-reported; r, Pearson correlation coefficient. Anxiety score, depression score, and neuroticism score were continuous phenotypic values of the residuals from linear regression models, while anxiety self and depression self were continuous phenotypic values of the residuals from logistic regression models. Anxiety score was defined according to the 7-item general anxiety disorder scale (GAD-7). Depression score was defined according to the Patient Health Questionnaire (PHQ-9). Neuroticism score was measured using the Eysenck Personality Questionnaire, and Revised Short Form (FPQ-R-S). The detailed description of neuroticism, anxiety and depression are shown in Additional file 1.

In total samples, one significant association were observed in plasma (Bonferroni-adjusted *P* value threshold: 3.29 × 10^− 4^), plasma protease C1 inhibitor (C1-Esterase Inhibitor) vs. neuroticism score (r = -0.01, *P* = 2.56 × 10^− 9^) (Fig. 1). In CSF (Bonferroni-adjusted *P* value threshold: 2.30 × 10^− 4^), five significant association were observed such as C1-Esterase Inhibitor vs. neuroticism score (r = -0.01, *P* = 3.09 × 10^− 8^), and NADPH-cytochrome P450 reductase (NADPH-P450 Oxidoreductase) vs. neuroticism score (r = -0.008, *P* = 2.51 × 10^− 5^) (Fig. 1).

### The gender characteristics of disease/trait-associated proteins

**Fig. 2:**
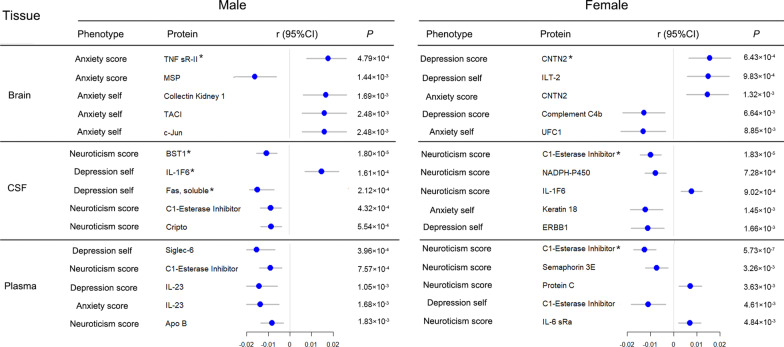
The top 5 correlations between protein and anxiety, depression and neuroticism in male and female. Dots indicate protein specific betas; Horizontal lines represent 95% CI. * indicates the protein that has reached the significant threshold of Bonferroni correction (7.14 × 10^− 4^ for brain, 3.29 × 10^− 4^ for plasma, and 2.30 × 10^− 4^ for CSF). self, self-reported; r, Pearson correlation coefficient. Anxiety score, depression score, and neuroticism score were continuous phenotypic values of the residuals from linear regression models, while anxiety self and depression self were continuous phenotypic values of the residuals from logistic regression models. Anxiety score was defined according to the 7-item general anxiety disorder scale (GAD-7). Depression score was defined according to the Patient Health Questionnaire (PHQ-9). Neuroticism score was measured using the Eysenck Personality Questionnaire, and Revised Short Form (FPQ-R-S). The detailed description of neuroticism, anxiety and depression are shown in Additional file 1.

In male participants of the UK Biobank cohort, 3 significant association was observed in CSF, such as ADP-ribosyl cyclase/cyclic ADP-ribose hydrolase 2 (BST1) vs. neuroticism score (r = -0.01, *P* = 1.80 × 10^− 5^), while there was no significant signal in plasma (Fig. 2). In female participants, 3 significant association were observed, including CNTN2 vs. depression score in brain (Bonferroni-adjusted *P* value threshold: 7.14 × 10^− 4^, r = 0.01, *P* = 6.43 × 10^− 4^), C1-Esterase Inhibitor vs. neuroticism score in CSF (r = -0.01, *P* = 1.83 × 10^− 5^) and plasma (r = -0.01, *P* = 5.73 × 10^− 7^) (Fig. 2).

### Differences in disease/trait-associated proteins in brain, cerebrospinal fluid, and plasma

**Fig. 3:**
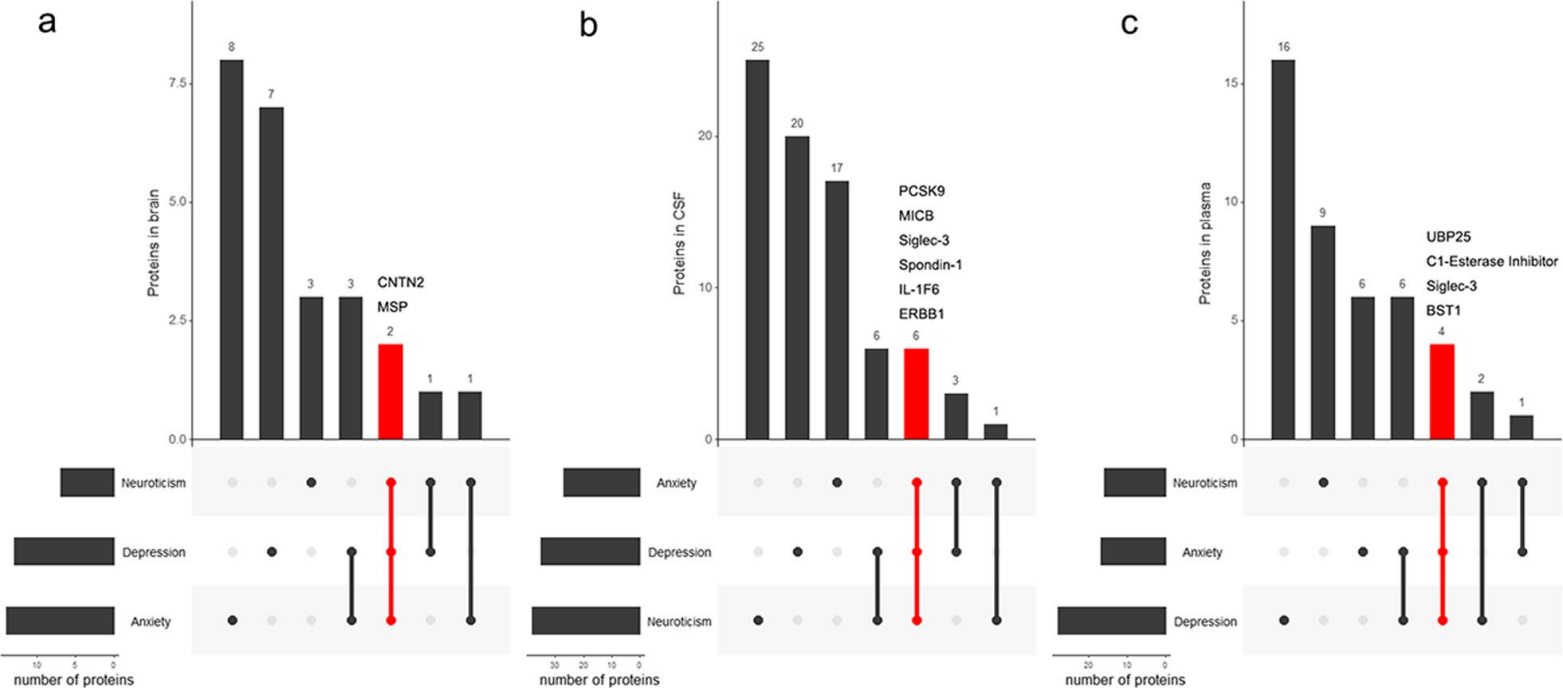
Differences in disorder-related proteins in different tissues. UpSet plot for all proteins related to psychiatric disorders. Red strip shows the common proteins among anxiety, depression and neuroticism. Dots and lines represent subsets of proteins. The protein types corresponding to the dots are contained in the subtype. The histogram represents the number of proteins in each subset. **a** Proteins in brain. **b** Proteins in CSF. **c** Proteins in plasma. *CSF* cerebrospinal fluid

After combining the candidate proteins (*P* < 0.05) of anxiety score and self-reported anxiety, depression score and self-reported depression, respectively, we obtained 15, 17 and 29 candidate proteins for anxiety in brain, plasma and CSF; 13, 29 and 38 candidate proteins for depression in brain, plasma and CSF; 6, 15 and 37 candidate proteins for neuroticism in brain, plasma and CSF, respectively (Fig. 3).We detected several disorder-specific and common proteins among the three traits. For example, human chorionic gonadotropin (r = -0.007, *P* = 1.86 × 10^− 2^) and luteinizing hormone (r = − 0.007, *P* = 1.95 × 10^− 2^) were associated only with anxiety in plasma. Copine-1 (r = 0.007, *P* = 1.13 × 10^− 2^) and complement C4b (r = − 0.007, *P* = 1.31 × 10^− 2^) were associated only with depression in brain. Plasminogen (r = -0.004, *P* = 4.26 × 10^− 2^) and macrophage-capping protein (r = − 0.005, *P* = 7.22 × 10^− 3^) were associated only with neuroticism in CSF. C1-Esterase Inhibitor, BST1, UBP25 and Siglec-3 were associated with all the three traits in plasma.

### Validation of common disease/trait-associated proteins in independent proteomic studies

To confirm the validity of our analysis, we validated the candidate proteins in our results that were commonly associated with neuroticism, depression and anxiety in other independent omics studies. As shown in Table 2, a total of 6 candidate proteins in our study have association signals in independent proteomic studies. For example, Contactin-2 (CNTN2) and Hepatocyte growth factor-like protein (MSP) were significantly correlated with anxiety and depression in the study of mouse proteomes by combining mass spectrometry. Ubiquitin carboxyl-terminal hydrolase 25 (UBP25) and Proprotein convertase subtilisin/kexin type 9 (PCSK9) were associated with neuroticism in human isobaric tags for relative and absolute quantification (iTRAQ)-based quantitative proteomic analysis. C1-esterase Inhibitor was significantly down-regulated in human CSF proteome of depression vs. control study.

**Table 2:** Association signals of common psychiatric disorder-related proteins in other proteomic studies

Protein	UniProt	Species	*P*_anxiety_	*P*_depression_	*P*_neuroticism_	Design
CNTN2	Q02246	Rat	0.087	0.019	/	Proteomes by combining mass spectrometry [66]
MSP	P26927	Rat	4.82 × 10^− 7^	1.23 × 10^− 6^	/	
UBP25	Q9UHP3	Human	/	/	0.540	Proteomic [67]
PCSK9	Q8NBP7	Human	/	/	0.431	
C1–Esterase Inhibitor	P05155	Human	0.021	0.021	/	Proteomic [68]
C1–Esterase Inhibitor	P05155	Human	/	< 0.05	/	CSF proteome analysis [69]
ERBB1	P00533	Human	/	6.52 × 10^− 6^	/	Combining GWAS and pQTL data [70]

*CNTN2* contactin-2, *MSP* hepatocyte growth factor-like protein, *UBP25* ubiquitin carboxyl-terminal hydrolase 25, *PCSK9* proprotein convertase subtilisin/kexin type 9, *C1-Esterase Inhibitor* plasma protease C1 inhibitor, *ERBB1* Epidermal growth factor receptor, *CSF* cerebrospinal fluid

### Validation of disease/trait-associated pQTLs in independent proteomic studies

**Fig. 4:**
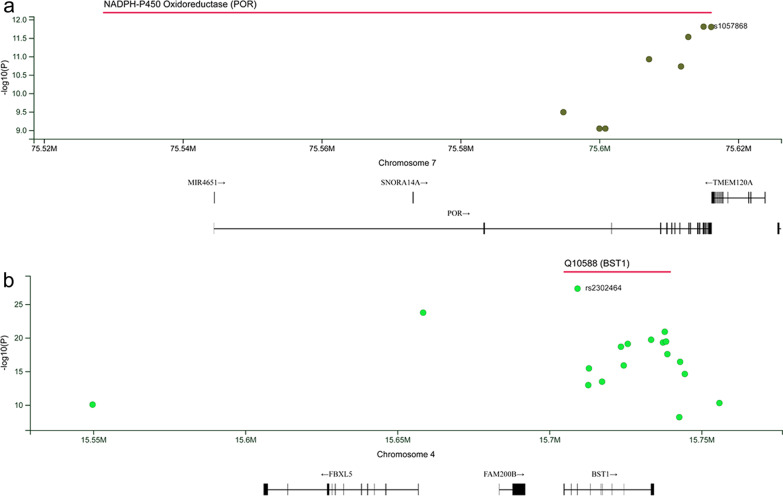
Variant-wise plots of pQTLs associated with phenotypes.The Y-axis represents the significance of pQTLs in the corresponding proteomic studies. The X-axis represents the chromosomal position. The black bars below the X-axis indicate the range of genes mapped to the corresponding chromosome location. The dots represent pQTL, while the red line represents the range of genes corresponding to pQTL. **a** NADPH-P450 Oxidoreductase (POR) has been detected in Alzheimer’s disease. **b** ADP-ribosyl cyclase/cyclic ADP-ribose hydrolase 2 (BST1) has been detected in schizophrenia, bipolar disorder, and depression

Our results were confirmed in other proteomic studies. As shown in Fig. 4, 8 pQTLs of NADPH-P450 Oxidoreductase (POR) were detected in the brain-spinal cord and linked to Alzheimer’s disease using genomic and multi-tissue proteomic integration. 18 pQTLs of BST1 were detected in the brain and linked to psychiatric disorders using genome-wide quantitative trait loci mapping of the human cerebrospinal fluid proteome such as depression. Detailed results of effectiveness evaluation are shown in Additional file 3.

## Discussion

In this study, we conducted a large observational and genetic PRS analyze to systematically evaluate the correlations between proteins and complex traits (anxiety, depression and neuroticism) using the UK Biobank cohort. We observed multiple significant correlations in plasma, CSF and brain. Further analysis provided evidence for gender differences between complex traits with protein in different tissues.

The proteomic dataset we used here is the largest brain and CSF pQTL analyses to date, as well as the first neurologically-relevant multi-tissue pQTL study, and a unique resource for leveraging multi-tissue pQTL to understand neurological traits [34]. The large sample sizes and well study design ensure the accuracy of tissue-specific protein identification. Understanding the tissue-specific genetic controls of protein level is essential to uncover mechanisms of post-transcriptional gene regulation. We used this proteomic dataset to explore the tissue-specific protein characteristics for anxiety, depression and neuroticism. Recent research confirmed that neurology and psychiatry both addressed disorders of the nervous system [41]. For example, psychiatric and neurologic depression seem to share common abnormalities and similar lesions in specific brain areas [42].

C1-Esterase inhibitor (C1-INH) in CSF and plasma were found to be significantly associated with neuroticism score in our study. The biologic activities of C1-INH may be divided into regulation of vascular permeability and anti-inflammatory functions [43]. We also found a negative correlation between C1-INH and neuroticism score. Recently, a number of studies confirmed the neuroprotection role of C1-INH that supports our results. For example, Mercurio et al. indicated that recombinant human C1-INH exhibited stronger neuroprotective effects than the corresponding plasma-derived protein after experimental ischemia/reperfusion injury in the brain [44]. Earlier studies found that C1-INH was produced in normal brain, whereas in Alzheimer disease (AD), C1-INH was significantly responsive to abnormal neuronal processes, such as dystrophic neurites and neuropil threads [45].

High neuroticism is a well-established risk for present and future depression and anxiety, as well as an emerging target for treatment and prevention [46]. It was notable that there were gender-specific proteins in samples with neuroticism. For the neuroticism-related proteins, BST1 was detected in males CSF, while C1-INH was detected in female plasma and CSF. BST1 is associated with the metabolism of nervous system and anxiety / depression-like behaviors. Higashida et al. tested *BST1* knockout mice of various ages to assess the relationship between the presence of *BST1* in the brain and its enzymatic activity, and indicated that BST1 might play a role in the embryonic and adult nervous systems [47]. After knocking out the *BST1*, the *BST1*^−/−^ male mice exhibited anxiety-related and depression-like behaviors compared with wild-type mice [48]. The SNP of *BST1* gene was found to be associated with multiple neurological and psychiatric conditions, including ASD, PD, and SCZ [49]. In addition, rs4698412 allele variant in *BST1* was shown to regulate lingual gyrus function and might be associated with brain activation and balance dysfunctions in PD [50].

Growing evidence highlights the similarities in psychoactive metabolites and microbiota-gut-brain axis among ASD, PD and AD. For example, psychobiotics are effective in improving neurodegenerative and neurodevelopmental disorders, including ASD, PD and AD [51]. The alterations in gut microbiome composition or diversity are implicated in the pathophysiology of neuropsychiatric disorders such as depression and anxiety, behavioural disorders such as ASD, and neurodegenerative disorders such as AD and PD [52]. Recent research confirmed that neurology and psychiatry both addressed disorders of the nervous system [41]. For example, psychiatric and neurologic depression seem to share common abnormalities and similar lesions in specific brain areas [42]. Depression and anxiety are common neuropsychiatric comorbidities of PD, and the somatic symptoms of depression often overlap with the motor symptoms of PD [53].

NADPH-P450 Oxidoreductase (POR) in CSF is another protein negatively associated with neuroticism score in our study. POR has a major role in metabolism of drugs and steroids [54]. Appropriate regulation of retinoic acid levels and tissue distribution by POR is essential for early embryonic development, brain morphogenesis and molecular patterning [55]. POR was detected in multiple brain areas. For example, immunohistochemistry test in rats showed that POR was located in the dopaminergic rich region of the periventricular hypothalamus and arcuate nucleus [56]. Haglund et al. found POR in the nigra, locus coereleus, dorsal raphe, hypothalamus, striatum, nucleus accumbens and olfactory tubercle [57]. Studies in the past decade have shown that POR could affect brain function and nervous system through the metabolism of nitric oxide [58], synapses forming [59], activity of Ca^2+^ channels [60], and cellular defense [61]. Together, these findings suggest that POR may relate to neuroticism by affecting brain function and neural development, but need more direct evidence.

Apolipoprotein L1 (APOL1) in plasma was found to be suggestively associated with self-reported depression in our study. APOL1 is ubiquitously expressed in human central nervous system (CNS), but at lower levels than that in peripheral tissues [62]. Situ hybridization studies also demonstrated pan-neuronal expression of *APOL1* mRNA in human frontal cortex [63]. Recent studies found that APOL1 was involved in brain structure and psychiatric disorders. For example, high-throughput molecular spectroscopy studies proved that APOL was an important factor in psychiatric disorders [64]. Hwang et al. used RNA-seq data from postmortem brain tissue hippocampus, and found that *APOL1* was one of the differentially up-regulated genes in patients with SCZ [65]. Hence, it can be inferred that APOL1 may induce abnormal function in the hippocampus, and may play a vital role in depression development.

This is the first systematic study of the relationship between proteins and depression / anxiety / neuroticism. However, our study does have certain limitations. The protein PRS data were collected from European ancestry, at the age averaged 82.2 years. The protein PRSs were calculated using the UK Biobank cohort, which were mainly middle-aged European populations. Thus, our findings should be carefully applied to young-aged and other ethnic populations. Besides, the pQTL dataset included both neurological disorder individuals and cognitively normal controls, which may result in slight bias on our results. Thirdly, since weight gain can be a symptom of depression, our adjustment for BMI in the regression analysis of depression phenotypes may have a potential bias for the results. In future studies, we need to validate our results using independent clinical samples or cohorts and explore the potential biological mechanism underlying the observed association between candidate proteins with depression, anxiety, and neuroticism.

Taken together, we systematically investigated the associations between proteins with depression, anxiety, and neuroticism utilizing UK Biobank individual level traits and genotype data and publicly available protein PRS data of brain, CSF, and plasma. Our study highlights the associations between neuroticism with C1-INH and POR, and may provide novel insights to uncover the roles of protein on the development of depression, anxiety and neuroticism.

## Supplementary Information

**Additional file 1. **Definitions of criterion for phenotypes in UK Biobank cohort

**Additional file 2. **Correlation matrix among the covariates and neuroticism, anxiety, and depression score

**Additional file 3. **Detailed results of effectiveness evaluation of protein PRS method in independent omics studies

## Data Availability

The datasets used and/or analysed during the current study are available from the corresponding author on reasonable request.

## Abbreviations

pQTL: Protein quantitative trait loci
CSF: Cerebrospinal fluid
PRS: Polygenic risk scores
eQTL: Expression quantitative trait locus
GWASs: Genome-wide association studies
TDI: Townsend deprivation index
BMI: Body mass index
SD: Standard deviation
SCZ: Schizophrenia
HDLs: High-density lipoproteins
ASD: Autism spectrum disorder
PD: Parkinson’s disease
