# Supplementary material for: A large-scale polygenic risk score analysis identified candidate proteins associated with anxiety, depression and neuroticism
Source: Mol Brain. 2022 Jul 23;15:66. doi: 10.1186/s13041-022-00954-3 (PMC9308259; doi:10.1186/s13041-022-00954-3)
Supplement: Supplementary file 1 — Additional file 1. Definitions of criterion for phenotypes in UK Biobank cohort [file 13041_2022_954_MOESM1_ESM.docx]

**Additional file 1**

***Definitions of criterion for neuroticism score***

Neuroticism score was defined according to neuroticism score from the UK Biobank fields 20127. Neuroticism score was measured using the Eysenck Personality Questionnaire, and Revised Short Form (FPQ-R-S)[1], which consists of 12 neurotic behavior domains as reported from the UK Biobank fields 1920, 1930, 1940, 1950, 1960, 1970, 1980, 1990, 2000, 2010, 2020 and 2030 from the touchscreen questionnaire at baseline. Participants were assessed for 12 domains of neurotic behaviors with a total score (0-12) by the touchscreen questionnaire, including: mood swings (1920), miserableness (1930), irritability (1940), sensitivity / hurt feelings (1950), fed-up feelings (1960), nervous feelings (1970), worrier / anxious feelings (1980), tense / 'highly strung' (1990), worry too long after embarrassment (2000), suffer from 'nerves' (2010), loneliness / isolation (2020), and guilty feelings (2030).

***Definitions of criterion for depression (depression score and self-report depression)***

Depression score was defined according to the Patient Health Questionnaire (PHQ-9)[2]. PHQ-9 is a classification algorithm with a total score (0-27) used to screen for and measure depression severity, focusing on nine depressive symptoms and signs (as detailed below：Little interest or pleasure in doing things 20514, Feeling down, depressed, or hopeless 20510, Trouble sleeping 20517, Feeling tired 20519, Poor appetite or overeating 20511, Feeling bad about yourself 20507, Trouble concentrating 20508, Moving or speaking slowly or fidgety or restless 20518, thoughts that you would be better off dead 20513).

In order to obtain a comprehensive and accurate control group of depressed and non-depressed patients, we self-reported depression, and strictly conducted the control group threshold by Davis et al. research[3], which based on Patient Health Questionnaire (PHQ-9)[2] and another strict criterion based on composite international diagnostic interview short-form (CIDI-SF)[2, 4]. Depression phenotype was defined according to three UK Biobank fields: 20002, 20126 and 20544. We selected participants who self-reported depression, based on the code 1286 from ID 20002, code 3,4 or 5 from ID 20126 and code 11 from ID 20544 as case.

And for the control of the depression, after excluding the self-reported depression defined in our study and depression single episode defined in Davis et al. research[3], we chose the participants who did not endorse depression or screen positive on PHQ[2] or CIDI-SF [4]. More precisely, participants whose PHQ score ≤ 5 and did not have core symptoms were selected. In detail, in order to meet the 0-3 scale for each question item in PHQ-9, the 9 symptom scores (1-4 scale) in the UKBB participants were all subtracted by one, which was then added up to create an overall sum score. The participants with PHQ ≤ 5 were selected as control for self-reported depression. According to CIDI-SF, core symptoms of depression were ID 20446 and ID 20441 in UK Biobank, we chose the participants who response “NO” to the question “Have you ever had a time in your life when you felt sad, blue, or depressed for two weeks or more in a row?” or “Have you ever had a time in your life lasting two weeks or more when you lost interest in most things like hobbies, work, or activities that usually give you pleasure?” as control for self-reported depression.

***Definitions of criterion for anxiety (anxiety score and self-report anxiety)***

Anxiety score was defined according to the 7-item general anxiety disorder scale (GAD-7)[5]. GAD-7 is a valid and efficient tool with a total score (0-27) for screening for generalized anxiety disorder and assessing its severity in clinical practice and research, focusing on seven anxious symptoms and signs (as detailed below: Feeling nervous, anxious or on edge 20506, Not being able to stop or control worrying 20509, Worrying too much about different things 20520, Trouble relaxing 20515, Being so restless that it is hard to sit still 20516, Becoming easily annoyed or irritable 20505, Feeling afraid as if something awful might happen 20512[5].

In order to obtain a comprehensive and accurate control group of anxiety and non-anxiety patients, we self-reported anxiety, and strictly conducted the control group threshold by Davis et al. research[3], which based on GAD-7. Anxiety phenotype was defined according to the UK Biobank fields 20002 and 20544. We selected participants who self-reported anxiety, based on the code 1287 from ID 20002, and code 15 from ID 20544 as case.

For the control of anxiety, after excluding the anxiety defined in our study and GAD ever defined in Davis et al. research[3], we chose the participants who did not endorse anxiety or screen positive on GAD-7 or CIDI-SF [4]. More precisely, participants with GAD score < 5 were selected for the control of the anxiety. In detail, in order to meet the 0-3 scale for each item of GAD, the 7 symptom scores (1-4 scale) in the UKBB participants were all subtracted by one, which was then added up to create an overall sum score. The participants with GAD score < 5 were selected as control for self-reported anxiety.

***Questionnaire wording and format***

Section A: Questions of depression in UK Biobank

Section B: Questions of anxiety in UK Biobank

Section C: Questions of neuroticism in UK Biobank

| **Section A: Questions of depression in UK Biobank** | | |
| --- | --- | --- |
| **Patient Health Questionnaire (PHQ-9) score:** | | |
| 1. **20514** 2. **20510** 3. **20534** 4. **20519** 5. **20511** 6. **20507** 7. **20508** 8. **20518** 9. **20513** | Over the last 2 weeks, how often have you been bothered by any of the following problems?  a. Little interest or pleasure in doing things  b. Feeling down, depressed, or hopeless  c. Trouble falling or staying asleep, or  sleeping too much  d. Feeling tired or having little energy  e. Poor appetite or overeating  f. Feeling bad about yourself or that you  are a failure or have let yourself or your  family down  g. Trouble concentrating on things, such  as reading the newspaper or watching  television  h. Moving or speaking so slowly that other  people could have noticed? Or the opposite — being so fidgety or restless that you have been moving around a lot more than usual  i. Thoughts that you would be better off  dead or of hurting yourself in some way | [Select one from the following for each of the statements]  - 01 Not at all  - 02 Several days  - 03 More than half the days  - 04 Nearly every day  - DA Prefer not to answer |
| \| **Core symptoms of depression based on CIDI** \| \| \| \| --- \| --- \| --- \| \| **20446** \| Have you ever had a time in your life when you felt sad, blue, or depressed for two weeks or more in a row? \| [Select one from]  - 01 Yes  - 00 No  - DA Prefer not to answer \| \| **20441** \| Have you ever had a time in your life lasting two weeks or more when you lost interest in most things like hobbies, work, or activities that usually give you pleasure? \| [Select one from]  - 01 Yes  - 00 No  - DA Prefer not to answer \|   **Section B: Questions of anxiety in UK Biobank** | | |
| **The 7-item anxiety scale (GAD-7) score** | | |
| 1. **20506** 2. **20509** 3. **20520** 4. **20515** 5. **20516** 6. **20505** 7. **20512** | Over the last 2 weeks, how often have you been bothered by any of the following problems?  a) Feeling nervous, anxious or on edge  b) Not being able to stop or control worrying  c) Worrying too much about different things  d) Trouble relaxing  e) Being so restless that it is hard to sit still  f) Becoming easily annoyed or irritable  g) Feeling afraid as if something awful might happen  [7 questions on one screen in grid] | [Select one from the following for each of the statements]  - 01 Not at all  - 02 Several days  - 03 More than half the days  - 04 Nearly every day  - DA Prefer not to answer |
| **GAD ever** | | |
| **20421** | Have you ever had a period lasting one month or longer when most of the time you felt worried, tense, or anxious? | - 01 Yes  - 00 No  - UN Do not know  - DA Prefer not to answer |
| **20420** | What is the longest period of time that this kind of worrying has ever continued? | Cbox2: Integer 0-99  Cbox1: Integer 0-11  Cbox02 & “year(s) and” & Cbox01 & “month(s)”  OR  - 03 All my life / as long as I can remember |
| **20425** | People differ a lot in how much they worry about things. Did you ever have a time when you worried a lot more than most people would in your situation? | - 01 Yes  - 00 No  - UN Do not know  - DA Prefer not to answer |
|  | Please think of the period in your life when you have felt worried, tense, anxious, or more worried than most people would in your situation. This could be in the past, or it could be continuing now. | Display throughout following questions C4 to C10 |
| **20542** | During that period, was your worry stronger than in other people? | - 01 Yes  - 00 No  - UN Do not know  - DA Prefer not to answer |
| **20538** | Did you worry most days? | - 01 Yes  - 00 No  - UN Do not know  - DA Prefer not to answer |
| **20543** | Did you usually worry about one particular thing, such as your job security or the failing health of a loved one, or more than one thing? | - 01 One thing  - 02 More than one thing  - UN Do not know  - DA Prefer not to answer |
| **20541** | Did you find it difficult to stop worrying? | - 01 Yes  - 00 No  - UN Do not know  - DA Prefer not to answer |
| **20540** | Did you ever have different worries on your mind at the same time? | - 01 Yes  - 00 No  - UN Do not know  - DA Prefer not to answer |
| **20539** | How often was your worry so strong that you couldn’t put it out of your mind no matter how hard you tried? | - 03 Often  - 02 Sometimes  - 01 Rarely  - 00 Never  - UN Do not know  - DA Prefer not to answer |
| **20537** | How often did you find it difficult to control your worry? | - 03 Often  - 02 Sometimes  - 01 Rarely  - 00 Never  - UN Do not know  - DA Prefer not to answer |
| 1. **20426** 2. **20423** 3. **20429** 4. **20419** 5. **20422** 6. **20417** 7. **20427** | When you were worried or anxious, were you also:  a) Restless?  b) Keyed up or on edge?  c) Easily tired?  d) Having difficulty keeping your mind on what you were doing?  e) More irritable than usual?  f) Having tense, sore, or aching muscles?  g) Often having trouble falling or staying asleep?  [Seven questions on one screen] | Force choice:  - 01 Yes  - 02 No  - NA Do not know  For following options: |
| **20428** | Did you ever tell a professional about these problems (medical doctor, psychologist, social worker, counsellor, nurse, clergy, or other helping professional)? | - 01 Yes  - 00 No  - UN Do not know  - DA Prefer not to answer |
| **20549** | Did you ever use the following for the worry or the problems it caused? (tick all that apply):  Please include any treatments that you have already told us about under ‘depression’ if they were also for anxiety: | - 01 Medication prescribed to you (for at least two weeks)  - 02 Unprescribed medication  (more than once)  - 03 Drugs or alcohol (more than once)  - 00 None of the above  - DA Prefer not to say |
| **20550** | Did you ever try talking therapies for these problems, or other structured activities you regard as therapeutic? Include only those you attended more than once.  Please include any treatments that you have already told us about under “depression” if they were also for anxiety: | [Select up to two]  - 01 Talking therapies, such as psychotherapy, counselling, group therapy or CBT  - 02 Other therapeutic activities such as mindfulness, yoga or art classes  - 00 None of the above  - DA Prefer not to answer |
| **20418** | Think about your roles at the time of this episode, including study / employment, childcare and housework, leisure pursuits. How much did these problems interfere with your life or activities? | [Choose one of]  - 03 A lot  - 02 Somewhat  - 01 A little  - 00 Not at all  - DA Prefer not to answer |
| **Section C: Questions of neuroticism in UK Biobank** | |  |
| **Neuroticism score base on Eysenck Personality Questionnaire, Revised Short Form (FPQ-R-S)** | | |
| 1. **1920** 2. **1930** 3. **1940** 4. **1950** 5. **1960** 6. **1970** 7. **1980** 8. **1990** 9. **2000** 10. **2010** 11. **2020** 12. **2030** | Work through these questions quickly and do not think  about the exact meaning of the question:   1. Does your mood often go up and down? 2. Do you ever feel 'just miserable' for no reason? 3. Are you an irritable person? 4. Are your feelings easily hurt? 5. Do you often feel 'fed-up'? 6. Would you call yourself a nervous person? 7. Are you a worrier? 8. Would you call yourself tense or 'highly strung'? 9. Do you worry too long after an embarrassing experience? 10. Do you suffer from 'nerves'? 11. Do you often feel lonely? 12. Are you often troubled by feelings of guilt? | [Select one from the following for each of the statements]  - 01 Yes  - 00 No  - DA Prefer not to answer |

**Reference**

1. Eysenck SBG, Eysenck HJ, Barrett P: A revised version of the psychoticism scale. *Personality & Individual Differences*, 6(1):21-29.

2. Kroenke K, Spitzer RL, Williams JBW, L?we B: The Patient Health Questionnaire Somatic, Anxiety, and Depressive Symptom Scales: a systematic review. 32(4):345-359.

3. Davis KAS, Cullen B, Adams M, Brailean A, Breen G, Coleman JRI, Dregan A, Gaspar HA, Hübel C, Lee W *et al*: Indicators of mental disorders in UK Biobank—A comparison of approaches. *International Journal of Methods in Psychiatric Research* 2019, 28(3):e1796.

4. Kessler RC, Andrews G, Mroczek D, Ustun B, Wittchen Hl: The World Health Organization Composite International Diagnostic Interview short‐form (CIDI㏒F). *International Journal of Methods in Psychiatric Research* 1998.

5. Spitzer RL, Kroenke K, Williams JB, Löwe B: A brief measure for assessing generalized anxiety disorder: the GAD-7. *Archives of internal medicine* 2006, 166(10):1092-1097.
